# Comparative Study of the Effects of Azilsartan and Telmisartan on Insulin Resistance and Metabolic Biomarkers in Essential Hypertension Associated With Type 2 Diabetes Mellitus

**DOI:** 10.7759/cureus.22301

**Published:** 2022-02-16

**Authors:** Bikash R Meher, Rashmi R Mohanty, Jyoti P Sahoo, Monalisa Jena, Anand Srinivasan, Biswa M Padhy

**Affiliations:** 1 Pharmacology, All India Institute of Medical Sciences, Bhubaneswar, Bhubaneswar, IND; 2 General Medicine, All India Institute of Medical Sciences, Bhubaneswar, Bhubaneswar, IND; 3 Pharmacology, Srirama Chandra Bhanja (SCB) Medical College and Hospital, Cuttack, IND

**Keywords:** biochemical markers, hypertension, diabetes mellitus, insulin resistance, telmisartan, azilsartan

## Abstract

Introduction

The complex interplay between the autonomic nervous system, renin-angiotensin-aldosterone system (RAAS), and immunity contributes to the pathogenesis of hypertension in diabetes mellitus. The objective of this study was to investigate and compare the effect of azilsartan and telmisartan on insulin resistance and metabolic biomarkers in patients with both hypertension and type 2 diabetes mellitus.

Methods

The present study was a prospective, randomized, active-controlled, open-label, parallel-group clinical trial. Patients with grade I or II essential hypertension with type 2 diabetes mellitus were randomized into two groups of 25 patients each. Baseline evaluation of homeostasis model assessment-insulin resistance (HOMA-IR), plasma glucose, insulin, leptin and adiponectin levels, and systolic and diastolic blood pressure (SBP and DBP) of patients was done. Patients were reassessed after 12 weeks of drug therapy with azilsartan 40 mg OD (once daily) or telmisartan 40 mg OD.

Results

The mean changes in HOMA-IR from the baseline at the end of 12 weeks of treatment were 0.15 (−0.64, 0.94.52) in the azilsartan group and 0.32 (−0.61, 1.26) in the telmisartan group. The mean difference in the changes from the baseline in HOMA-IR between the two groups was 0.3 (−0.87, 1.48), which was not statistically significant. No statistically significant changes were observed between the two groups in metabolic biomarkers (leptin: -0.84, CI: -4.83 to 3.14, and adiponectin: -0.12, CI: -0.62 to 0.37). Systolic (SBP) and diastolic blood pressure (DBP) decreased at the end of the 12-week treatment in both the groups; however, there was no significant difference between the two groups (SBP: -2.6, CI: -10.35 to 5.1, and DBP: -3.0, CI: -7.7 to 1.7).

Conclusion

Neither azilsartan nor telmisartan had any significant effects on insulin resistance and metabolic biomarkers after 12 weeks of drug therapy in hypertension patients associated with type 2 diabetes mellitus. However, they showed a comparable antihypertensive effect. The adverse effects observed were mild in nature, and their incidence was comparable between the two groups.

## Introduction

Epidemiological analysis suggests that hypertension is more prevalent among type 2 diabetes mellitus patients [1]. Furthermore, the coexistence of diabetes and hypertension increases the risk of microvascular and macrovascular complications [2]. Angiotensin II, an important hormone of the renin-angiotensin-aldosterone system (RASS), plays a crucial role in the pathogenesis of both hypertension and insulin resistance [3]. Drugs acting on RASS such as angiotensin-converting enzyme inhibitors (ACEI) and angiotensin II receptor blockers (ARBs) are preferred in the patients of hypertension associated with type 2 diabetes mellitus as they have the potential to reduce plasma glucose along with the reduction in blood pressure [4-6]. However, not all ARBs exhibit a similar effect on insulin resistance and cardiometabolic syndrome. Angiotensin II receptor blockers such as olmesartan and candesartan do not significantly improve insulin resistance in animal and human studies, whereas other ARBs such as telmisartan do so [7-12]. This difference in effect is probably attributed to the difference in their chemical structure and mechanism of action. Telmisartan act as a partial agonist to peroxisome proliferator-activated receptor-γ (PPAR-γ) and increases the activation of PPAR-γ target genes in preadipocyte fibroblasts, whereas other ARBs do not [13]. 

Azilsartan medomoxil is a relatively new addition to the class of ARBs approved for the indication of essential hypertension [14,15]. Evidence generated in the preclinical studies suggests that like telmisartan, azilsartan too improves insulin sensitivity in different animal models by affecting PPAR-γ and TNF-α and, hence, can be expected to promote insulin sensitivity in human subjects [16,17]. However, there is a paucity of data available for the head-on comparison between the effect of telmisartan and azilsartan on insulin resistance and metabolic biomarkers. Therefore, we planned to explore those effects by measuring the homeostasis model assessment ratio of insulin resistance (HOMA-IR) and plasma level of leptin and adiponectin.

## Materials and methods

The study was initiated following the approval from the Institutional Ethics Committee of All India Institute of Medical Sciences (AIIMS), Bhubaneswar (IEC AIIMS/BBSR/ T/IM-F/17-18/30). It was carried out following the ethical principles laid down by the Indian Council of Medical Research’s National Ethical Guideline for biomedical and health research involving human participants (2017).

Study design

The study was a 12-week randomized, parallel-group, open-label, active-controlled study with a 1:1 allocation ratio. Eligible patients were briefed regarding the objective of the study along with anticipated risks and benefits, and written informed consent was obtained from interested patients. Five milliliters of blood sample were collected from overnight fasted patients for the estimation of baseline serum insulin, glucose, leptin and adiponectin. Then, they were randomized by a simple randomization method into two groups using computer-generated random codes while ensuring allocation concealment. One group of patients received tablet telmisartan 40 mg once daily (OD), and another group received tablet azilsartan 40 mg OD for 12 weeks. Clinical and biochemical parameters were reassessed at the end of 12 weeks of treatment for both groups.

Selection of participants

Patients of either sex aged between 18 and 65 years who are attending general medicine outpatient department with hypertension (systolic < 180 mm of Hg and diastolic < 100 mm of Hg) along with type 2 diabetes mellitus, treatment-naive patients, or patients not taking antihypertensive treatment for at least one month before the commencement of study were included in the study. Patients with type 1 diabetes mellitus, complicated type 2 diabetes mellitus on hypoglycemic therapy other than metformin, using drugs acting on RAS (except telmisartan and azilsartan) within one month before the enrollment, and a history of allergy to azilsartan or telmisartan were excluded from the study. Patients with clinically evident renal dysfunction (serum creatinine 1.5 mg/dl) and hyperkalemia (potassium level ≥ 5.5 mEq), pregnant and lactating women, and patients with any serious hepatic, cardiovascular, or pulmonary conditions were also precluded from the study.

Primary and secondary outcomes

The primary outcome measure in this study was a change in the HOMA-IR value at the end of 12 weeks from the baseline. HOMA-IR was calculated by applying the formula: fasting insulin (μU/mL) × fasting glucose (mg/dL)/405. The secondary outcome measures were change in leptin, adiponectin, and blood pressure levels from baseline to 12 weeks of follow-up. The occurrence of treatment-emergent adverse events between the two groups was also compared.

Statistical analysis

All continuous variables were expressed as mean ± SD. Means of continuous variables were compared by the two-sided paired, unpaired t-test. Multivariate linear was used to negate the effects of drugs other than azilsartan and telmisartan on insulin sensitivity. P < 0.05 was considered significant. Statistical software SPSS version 22 (IBM Corp., Armonk, NY) was used for analysis. A sample size of 25 in each group is required to detect a logarithmic difference of 0.4 in HOMA-IR values between the two groups with 0.4 as the variance in each group. The power of the sample is 90%, which allows an alpha error of 0.05. As HOMA-IR values are exponentially distributed, logarithmic values are used for sample size calculation.

## Results

**Figure 1:**
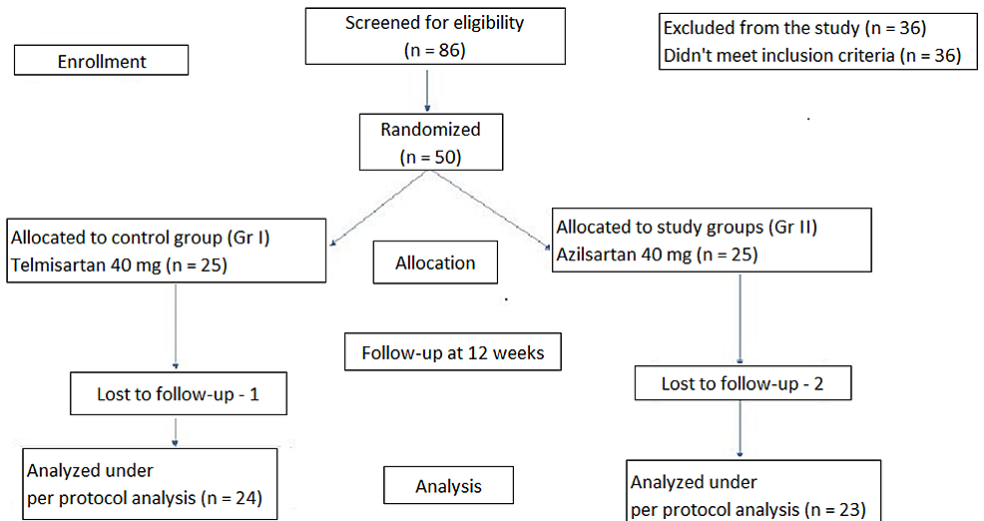
CONSORT diagram showing the flow of participants through each stage of randomized trial CONSORT: Consolidated Standards of Reporting Trials.

Eighty-six patients who were diagnosed with hypertension and type 2 diabetes mellitus were screened for the study, out of which 36 patients were excluded as they did not satisfy the inclusion criteria. One patient in the telmisartan group and two patients in the azilsartan group were lost to follow-up (Figure 1).

Patient demographic and baseline characteristics

Table 1 shows the baseline characteristics of the study participants. At baseline, there were no significant differences in age, gender, biochemical, and clinical parameters between the two groups.

**Table 1:** Baseline demographic data and clinical characteristics of the study participants HOMA-IR: Homeostasis model assessment-insulin resistance; SBP: systolic blood pressure; DBP: diastolic blood pressure.

Characteristics	Telmisartan 40 mg	Azilsartan 40 mg	P-value
No. of patients recruited	25	25	
No. of patients who completed the study	24	23	
Mean age in years	54.42 ± 7.83	53.09 ± 8.02	0.89
Male:female ratio	14:10	13:10	
HOMA-IR	4.5 ± 0.35	5.04 ± 0.25	0.22
Leptin (ng/ml)	12.91 ± 1.45	13.97 ± 2.6	0.82
Adiponectin (µg/ml)	5.5 ± 0.16	5.65 ± 0.13	0.48
SBP (mm of Hg)	151 ± 2.72	156 ± 3.1	0.66
DBP (mm of Hg)	85 ± 1.96	87 ± 1.32	0.72

Change in HOMA-IR

The mean HOMA-IR value at baseline was 4.5 (±0.35) for the telmisartan group and 5.04 (±0.25) for the azilsartan group, and there was no significant difference in the baseline value between the two groups (p = 0.229). The change in HOMA-IR from the baseline at the end of 12 weeks’ treatment was 0.32 (95% CI: −0.61 to 1.26) and 0.15 (95% CI: −0.64 to 0.94) in the telmisartan and azilsartan group, respectively. Moreover, no significant changes in HOMA-IR were observed between the two groups at the end of 12 weeks (0.3; 95% CI: -0.87 to 1.48, p > 0.05).

Change in plasma leptin

The mean plasma leptin level (ng/ml) at baseline was 12.91 ± 1.45 and 13.27 ± 2.6 for telmisartan and azilsartan groups, respectively, and the difference between the groups was recorded as not significant (p = 0.825). No significant change was observed in the serum leptin levels from baseline to 12 weeks in both the treatment groups, and the difference in change between the two groups at 12 weeks was also not statistically significant (-0.84; 95% CI: -4.83 to 3.14, p > 0.05) (Table 2).

**Table 2:** Change in the efficacy parameters in the study groups over a period of 12 weeks (per-protocol analysis) HOMA-IR: Homeostasis model assessment-insulin resistance; SBP: systolic blood pressure; DBP: diastolic blood pressure.

Variables	Telmisartan 40 mg (n = 24)				Azilsartan 40 mg (n = 23)				Between the two groups at 12 weeks	
	Baseline	Follow-up	Mean difference	P-value	Baseline	Follow-up	Mean difference	P-value	Mean difference	P-value
HOMA-IR	4.5 ± 0.35	4.18 ± 0.41	0.32 (-0.61 to 1.26)	0.48	5.04 ± 0.25	4.89 ± 0.32	0.15 (-0.64 to 0.94)	0.69	0.3 (-0.87 to 1.48)	0.60
Leptin (ng/ml)	12.91 ± 1.45	13.15 ± 1.38	-0.24 (-1.81 to 1.34)	0.75	13.27 ± 2.6	12.64 ± 2.46	0.63 (-3.1 to 4.36)	0.72	-0.84 (-4.83 to 3.14)	0.67
Adiponectin (µg/ml)	5.5 ± 0.16	5.4 ± 0.16	.044 (-0.36 to 0.45)	0.82	5.65 ± 0.13	5.49 ± 0.19	0.16 (-0.14 to 0.47)	0.28	-0.12 (-0.62 to 0.37)	0.63
SBP (mm of Hg)	151 ± 2.72	135 ± 2.83	15.75 (9.65 to 21.8)	<0.05	156 ± 3.1	138 ± 3.4	18.26 (13.03 to 23.4)	<0.05	-2.6 (-10.35 to 5.13)	0.50
DBP (mm of Hg)	85 ± 1.96	79 ± 1.65	5.2 (1.77 to 8.77)	<0.05	87 ± 1.32	78 ± 1.29	8.69 (5.68 to 11.7)	<0.05	-3.0 (-7.7 to 1.7)	0.20

Change in plasma adiponectin

The mean plasma adiponectin level(µg/ml) at baseline was 5.5 ± 0.16 and 5.65 ± 0.13 for telmisartan and azilsartan groups, respectively, and there was no significant difference in the baseline value between the two groups (p = 0.486). We did not observe any significant change in the serum adiponectin levels in both the groups over the period of 12 weeks’ treatment with both telmisartan and azilsartan. The difference in change between the two groups was also compared and found to be statistically not significant (-0.12; 95% CI: -0.62 to 0.37, p > 0.05) (Table 2).

Change in blood pressure

The mean systolic blood pressure (SBP) values at baseline were 151 ± 2.72 and 156 ± 3.1 mm of Hg for telmisartan and azilsartan, respectively. The difference in baseline values between the two treatment groups was not statistically significant (p = 0.661). There was a significant decrease in SBP in both the treatment groups (telmisartan: 15.75; 95% CI: 9.65 to 21.8, p < 0.05; and azilsartan: 5.2; 95% CI: 1.77 to 8.77, p < 0.05), but the decrease was more in the azilsartan group than the telmisartan group. However, the difference between the two groups was not statistically significant (95% CI: -10.35 to 5.13). Similarly, the mean clinic diastolic blood pressure (DBP) changes were also decreased significantly in both the groups (telmisartan: 5.2; 95% CI: 1.77 to 8.77, p < 0.05; and azilsartan: 8.69; 95% CI: 5.68 to 11.7, p < 0.05), and the difference between the two groups was not statistically significant (95% CI: -7.7 to 1.7) (Table 2).

Safety evaluation

Out of 47 patients, 30 (63%) patients had more than one adverse event during the 12-weeks treatment period. The incidence of adverse events was 14 (58%) with telmisartan and 16 (69%) with azilsartan. The most common adverse events reported were headaches, back pain, and a decrease in blood pressure. On the basis of the modified Hartwig and Siegel scale of severity assessment, all the adverse events were categorized as mild (level 1) in severity.

## Discussion

Angiotensin receptor blockers are recommended as the initial choice of treatment in hypertension patients with type 2 diabetes mellitus on account of its pleiotropic effects apart from its primary antihypertensive action [18-20]. Previous trials have evaluated and compared the effect of ARBs in such patients with respect to control of blood pressure and improvement in insulin resistance. In our study, we compared the effects of telmisartan and azilsartan on HOMA-IR, which is a surrogate marker for estimating insulin resistance. Changes in mean HOMA-IR value from the baseline to the end of 12-week treatment were 0.32 (95% CI: −0.61 to 1.26) in the telmisartan group and 0.15 (95% CI: −0.64 to 0.94) in the azilsartan group, thus implying that insulin resistance did not remarkably improve in either azilsartan or telmisartan group, which was in accordance with the study done by Naruse et al. [21]. Similarly, a pooled analysis comparing telmisartan with other ARBs noted no significant effect on HOMA-IR in hypertensive patients with insulin resistance or diabetic states [6]. However, a meta-analysis performed on randomized trials of telmisartan versus active control for insulin resistance in hypertensive patients showed a significant improvement in the insulin resistance by telmisartan [22].

In this study, we also assessed the change in serum biomarkers such as adiponectin and leptin level over 12 weeks of treatment with telmisartan and azilsartan. Leptin and adiponectin are differentially expressed adipokines in obesity and cardiovascular diseases. Leptin levels are directly associated with adipose tissue mass, while adiponectin levels are downregulated in obesity [23]. Contrasting pieces of evidence are associated with the effect of ARB blockers on adiponectin and leptin level in human subjects as well. Few studies have demonstrated a significant change in the level of serum biomarkers with the use of telmisartan, whereas other studies did not record any such changes following the use of telmisartan. Adiponectin is an abundantly expressed adipokine that exerts a potent insulin-sensitizing effect. In a study by Kubik et al., six months’ treatment with telmisartan 40 mg OD significantly increased the adiponectin value in obese patients with arterial hypertension [24]. A study by Mori et al. demonstrated an increase in the adiponectin level with a high dose (80 mg) of telmisartan in patients with diabetes and hypertension [25]. However, in a study by de Luis et al. evaluating the effect of telmisartan in hypertensive obese patients, telmisartan did not alter the adiponectin level [8]. Our study did not reveal any significant changes in serum adiponectin following treatment with telmisartan and azilsartan.

In this study, we also assessed the change in serum leptin level over 12 weeks’ treatment with either telmisartan or azilsartan. There was not any significant change recorded in both the groups after the follow-up period of 12 weeks. However, the serum leptin level was significantly increased in three months of telmisartan treatment in the study performed by Usui et al. in patients having hypertension and diabetes mellitus [26]. These findings suggest that apart from the known mechanism of action, some hitherto unknown factors might also sway the effect of ARBs in improving insulin resistance in patients with hypertension-associated type 2 diabetes mellitus.

Both azilsartan and telmisartan administration for 12 weeks resulted in a significant reduction in both DBP and SBP from baseline in this study. However, no significant difference was observed between the two groups. Azilsartan and telmisartan both demonstrated favorable safety and tolerability profiles in our patient population, and we did not identify any new safety signals.

Our study has some inherent limitations. It was an open-label study; so, the chances of bias may be more. It was carried out in a small population and for a relatively short follow-up period for a chronic disease like diabetes mellitus. This may have played a role in some of the negative findings.

## Conclusions

From this study, we conclude that both azilsartan and telmisartan had no statistically significant effect on HOMA-IR, which is a well-known predictor of insulin resistance after 12 weeks of therapy. Also, both the drugs did not show any significant effect on leptin and adiponectin. However, both SBP and DBP were reduced significantly following the 12 weeks’ therapy. The adverse effects observed were mild in nature, and their incidence was comparable between the two groups. Further studies are required to elucidate the long-term effects of azilsartan and telmisartan in hypertension patients with type 2 diabetes mellitus.
